# Sublingual Immunotherapy Induces Regulatory Function of IL-10-Expressing CD4**^+^**CD25**^+^**Foxp3**^+^** T Cells of Cervical Lymph Nodes in Murine Allergic Rhinitis Model

**DOI:** 10.1155/2012/490905

**Published:** 2012-10-17

**Authors:** Takaya Yamada, Miki Tongu, Kaoru Goda, Noriaki Aoi, Ichiro Morikura, Takafumi Fuchiwaki, Hideyuki Kawauchi

**Affiliations:** ^1^Department of Experimental Animals, Center for Integrated Research in Science, Shimane University, 89-1 Enya-cho, Izumo, Shimane 693-8501, Japan; ^2^Department of Otorhinolaryngology, Faculty of Medicine, Shimane University, 89-1 Enya-cho, Izumo, Shimane 693-8501, Japan

## Abstract

Sublingual immunotherapy (SLIT) has been considered to be a painless and efficacious therapeutic treatment of allergic rhinitis which is known as type I allergy of nasal mucosa. Nevertheless, its mechanisms need to be further investigated. In this study, we constructed an effective murine model of sublingual immunotherapy in allergic rhinitis, in which mice were sublingually administered with ovalbumin (OVA) followed by intraperitoneal sensitization and nasal challenge of OVA. Sublingually treated mice showed significantly decreased specific IgE responses as well as suppressed Th2 immune responses. Sublingual administration of OVA did not alter the frequency of CD4^+^CD25^+^ regulatory T cells (Tregs), but led to upregulation of Foxp3- and IL-10-specific mRNAs in the Tregs of cervical lymph nodes (CLN), which strongly suppressed Th2 cytokine production from CD4^+^CD25^−^ effector T cells *in vitro*. Furthermore, sublingual administration of plasmids encoding the lymphoid chemokines CCL19 and CCL21-Ser DNA together with OVA suppressed allergic responses. These results suggest that IL-10-expressing CD4^+^CD25^+^Foxp3^+^ Tregs in CLN are involved in the suppression of allergic responses and that CCL19/CCL21 may contribute to it in mice that received SLIT.

## 1. Introduction

Allergic rhinitis is a Th2-mediated disorder characterized by antigen-specific IgE production and several nasal symptoms such as sneezing, nasal congestion, itching, and rhinorrhea [1]. Crosslinking of the IgE-Fc*ε* receptor complexes by allergen leads to the degranulation of mast cells and basophils, so that histamine and other allergy-associated chemical mediators are released for the immediate phases of allergic responses. Chemokines such as CCL5 (RANTES), CCL11 (eotaxin), CCL17 (thymus and activation-regulated chemokine), and CCL22 (macrophage-derived chemokine) are released by mast cells and other cells and trigger recruitment of inflammatory cells including eosinophils and Th2 cells for the induction of late-phase allergic responses [2, 3].

Allergen-specific immunotherapy has been employed to redirect inappropriate immune responses in patients with allergic rhinitis [4]. Subcutaneous inoculation of allergen has been shown to modulate allergen-specific T cell and B-cell responses and reduce the recruitment and activation of proinflammatory cells in the target mucosa [5]. Subcutaneous immunotherapy (SCIT) significantly reduces symptoms and medication requirements in patients with allergic rhinitis but its use is limited by the possibility of severe systemic reactions [6]; therefore sublingual administration of allergen is raising interest as an alternative route for allergen delivery. Numerous clinical trials have shown that sublingual immunotherapy (SLIT) in patients with allergic rhinitis is clinically effective in terms of symptoms and medication requirements [6]. Although recent studies found regulatory T cells (Tregs) to be involved in the suppression of allergic responses in SLIT [7], their mechanisms of action have not been fully elucidated.

The CCR7 ligands CCL19 and CCL21 are lymphoid chemokines involved in the chemotaxis of lymphoid cells such as leukocytes and dendritic cells (DCs) and in the formation of appropriate cellular microcompartmentalization and homeostasis in lymphoid tissues [8]. CCL21 is encoded by two genes, *Scya21a* (CCL21-Ser) and *Scya21b* (CCL21-Leu) [9]. Paucity of lymph node T cells (*plt*) mice has a genomic deletion that includes the CCL19/CCL21-Ser gene, leading to defective homing of naïve T cells to the secondary lymphoid tissues and thereby to insufficient architectural development of nasopharynx-associated lymphoid tissue (NALT) and of other organized lymphoid tissues such as spleen and cervical lymph nodes (CLN) [8–10]. The CCR7-CCL19/CCL21 signaling pathway is also essential for the induction of antigen-specific immune responses to sublingually administered antigen [11]. We previously reported that the CCR7-CCL19/CCL21 signal is responsible for the migration of CD4^+^CD25^+^ Tregs into CLN and NALT and for the suppression of excessive Th2 response in allergic state [12]. Nasal administration of plasmids encoding CCL19/CCL21-Ser-DNA suppressed allergic responses in the murine allergic rhinitis model [12].

In the current study, we constructed two models of SLIT, that is, preventive SLIT model in which mice were sublingually treated with allergen before systemic and local sensitization and therapeutic SLIT model in which mice received SLIT after systemic sensitization and nasal allergen challenge in allergic rhinitis. We demonstrated suppressed antigen-specific Th2-mediated allergic responses and upregulation of Foxp3 and IL-10 gene in CD4^+^CD25^+^ Tregs of CLN in mice treated with preventive SLIT. Our results suggest that Tregs in CLN are involved in the suppression of Th2-mediated allergic responses in mice received SLIT. Moreover, in mice prophylactically treated by SLIT with antigen together with plasmid encoding CCL21-Ser-DNA, Th2 cytokine expression was suppressed.

## 2. Materials and Methods

### 2.1. Mice

Female BALB/c mice were purchased from Japan SLC (Hamamatsu, Japan). These mice were maintained under specific pathogen-free conditions and received OVA-free diet in the laboratory animal research center of Shimane University. All mice were 6-7 weeks of age at the beginning of individual experiments.

### 2.2. Induction of Allergic Rhinitis

For the induction of allergic rhinitis, we employed a previously described protocol with some modifications in the quantity of antigens and the sensitization schedule [12]. In brief, BALB/c mice were presensitized by means of an intraperitoneal injection of 25 *μ*g of ovalbumin (OVA) (Grade V; Sigma Chemical Co., St. Louis, MO, USA) with 1 mg of aluminum hydroxide hydrate gel (Alum) (LSL Co., Tokyo, Japan) in 200 *μ*L of PBS once a week for three weeks. Thereafter, mice were challenged by nasal administration of 400 *μ*g of OVA in 4 *μ*L of PBS for 14 consecutive days.

### 2.3. Sublingual Treatment

Mice were anesthetized by intraperitoneal injection of sodium pentobarbital (50 mg/kg) and 4 *μ*L of PBS alone or PBS containing 500 *μ*g of OVA that was applied on sublingual mucosa under the tongue as previously described with some modifications [13]. Mice were held with their heads placed in a horizontal position for 20 minutes to prevent them from swallowing the antigen.

### 2.4. ELISA for the Analysis of Antigen-Specific IgE

OVA-specific IgE level in serum was analyzed using DS mouse IgE ELISA (OVA) (DS Pharma Biomedical, Osaka, Japan).

### 2.5. Isolation of Mononuclear Cells

Spleen and CLN were removed and single-cell suspensions were prepared by mechanical dissociation [14]. Single cells of NALT and nasal passage (NP) were isolated as previously described with some modifications [14]. In brief, the palatine plate containing NALT was removed and then NALT was dissected out. NP tissues without NALT were also extracted from the nasal cavity, and mononuclear cells from individual tissues were isolated by gentle teasing using needles through a 40 *μ*m nylon mesh.

### 2.6. Analysis of Cytokine Production by CD4^+^ T Cells

For the purification of CD4^+^ T cells, isolated mononuclear cells were incubated with CD4 (L3T4) MicroBeads (Miltenyi Biotec, Bergisch Gladbach, Germany) at 4°C for 30 minutes. CD4^+^ cells were sorted by VarioMACS Separator (Miltenyi Biotec) and suspended in complete RPMI medium containing 10% FBS, 5 *μ*M 2 ME, 10 U/ml of penicillin, and 100 *μ*g/ml of streptomycin. Cells were then cultured at a density of 1 × 10^5^ cells/well in the presence of 1 mg/ml of OVA with splenic feeder cells (5 × 10^5^ cells/well) which were pretreated with mitomycin−C (Sigma Chemical Co.) in round-bottom 96-well microculture plates for 72 hours [15]. Culture supernatants were collected and examined for the production of cytokines (IL-4, IL-5, IL-13, and IFN-*γ*) by cytokine ELISA kits (R&D Systems Inc., Minneapolis, MN, USA).

### 2.7. Flow Cytometric Analysis

For the flow cytometric analysis, single cells isolated from several tissues were first incubated with anti-CD16/CD32 (2.4G2; BD Biosciences, San Jose, CA, USA) to block nonspecific binding of antibodies to the Fc*γ*III and Fc*γ*II receptors and then stained with each antibody. Cells were stained with fluorescein-isothiocyanate(FITC-) conjugated anti-CD4 (L3T4; BD Biosciences) and phycoerythrin-(PE-) conjugated anti-CD25 (PC61; BioLegend, San Diego, CA, USA). Compensation was carefully performed in each tissue in accordance with the published instructions [16]. Stained cells were then analyzed using an EPICS XL flow cytometer (Beckman Coulter Inc., Fullerton, CA, USA).

### 2.8. Reverse Transcription PCR

Total RNA was extracted using TRIzol reagent (Invitrogen, Carlsbad, CA, USA) and cDNA was obtained by reverse transcription using High-Capacity cDNA Reverse Transcription Kit (Applied Biosystems, Foster, CA, USA). Semiquantitative PCR was performed using commercial primer of mouse GAPD, Foxp3, and IL-10 (Applied Biosystems) and designed primers (Nihon Gene Research Laboratories, Sendai, Japan) as follows: CCL19, forward, 5′-CCATCCCTGGGAACATCGTG-3′ and reverse, 5′-TTCGGATGATGCGATCCACCC-3′;CCL21-Ser, forward, 5′-GGAAGCACTCTAAGCCTGAGC-3′ and reverse, 5′-CTTTCCAGACTTAGAGGTTCCC-3′. The reactions were amplified for 30 cycles and analyzed on 2% agarose gels stained with ethidium bromide.

### 2.9. Quantitative Real-Time PCR

Quantitative real-time PCR was performed using ABI PRISM 7700 (Applied Biosystems) with commercial primers (Foxp3, IL-10, TGF-*β*, IL-4, IL-5, and IL-13) for TaqMan Gene Expression Assays (Applied Biosystems). Messenger RNA expression levels for specific genes were normalized as a ratio relative to GAPD.

### 2.10. T-Cell Suppression Assay

CD4^+^ T cells were purified from single cells isolated from CLN by magnetic separation. Thereafter, CD4^+^CD25^+^ Tregs were separated as a positive fraction by using CD4^+^CD25^+^ Regulatory T-Cell Isolation Kit (Miltenyi Biotec) and CD4^+^CD25^−^ cells as a negative fraction. CD4^+^CD25^−^ cells isolated from CLN of mice which developed allergic rhinitis without having sublingual treatment were incubated with CD4^+^CD25^+^ cells isolated from CLN of mice sublingually treated with either PBS or OVA at the ratio of 1 : 1 in the presence of feeder cells and plate-bound anti-CD3*ε* (145-2C11; BioLegend) and soluble anti-CD28 (37.51; BioLegend) antibodies for 96 hours. Cytokine levels in culture supernatants were examined by cytokine ELISA kit.

### 2.11. Construction of Plasmid Encoding CCL19/CCL21-Ser cDNA

Plasmid DNA encoding either CCL19 or CCL21-Ser was constructed as previously described [12]. Briefly, CCL19, and CCL21-Ser cDNAs were amplified by PCR using cDNA from whole spleen cells of naïve BALB/c mice as a template. Plasmid DNA encoding either CCL19 or CCL21-Ser was constructed by the ligation of CCL19 or CCL21-Ser cDNA, respectively, into a pIRES2-EGFP vector (BD Biosciences Clontech, Palo Alto, CA, USA). The empty vector pIRES2-EGFP (mock DNA) was used as a control. The plasmid DNAs and the mock DNA were amplified in *Escherichia coli* and purified using an EndoFree Plasmid Maxi Kit (Qiagen, Valencia, CA, USA).

### 2.12. Statistical Analysis

Data were expressed as mean ± SE and evaluated by either an unpaired Student's *t*-test for comparing the means of two independent groups or Kruskal-Wallis test for comparing more than three independent groups. *P* values <0.05 were assumed to be statistically significant.

## 3. Results

### 3.1. Sublingual Administration of OVA before Systemic Sensitization Suppressed OVA-Specific IgE Production in Serum

**Figure 1:**
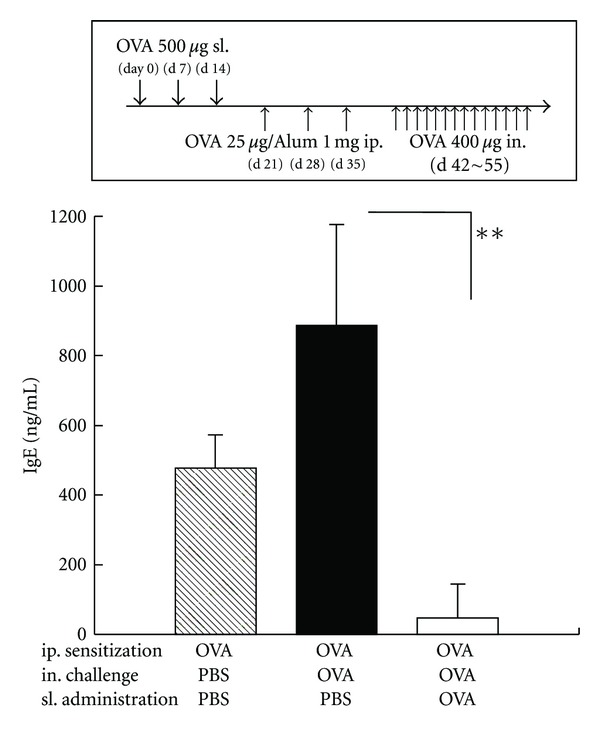
Antigen-specific allergic responses in sublingually treated mice with allergic rhinitis. Mice were sublingually administered with PBS alone or OVA followed by intraperitoneal sensitization and nasal challenge with OVA. OVA-specific IgE levels in serum were assayed by sandwich ELISA. The data are representative of three independent experiments containing three to five mice in each group. Significance was evaluated by Kruskal-Wallis test. **P < 0.01.

Mice sublingually administered with OVA before systemic sensitization and nasal challenge showed significantly decreased level of serum antigen-specific IgE compared to control mice which were sublingually treated with PBS (Figure 1).

We also examined OVA-specific IgE levels in the respiratory mucosal compartment (i.e., nasal wash and bronchoalveolar lavage), although those were undetectable (data not shown).

### 3.2. Th2 Cytokine Production by T Cells of Spleen Was Suppressed after Sublingual Administration of OVA

**Figure 2:**
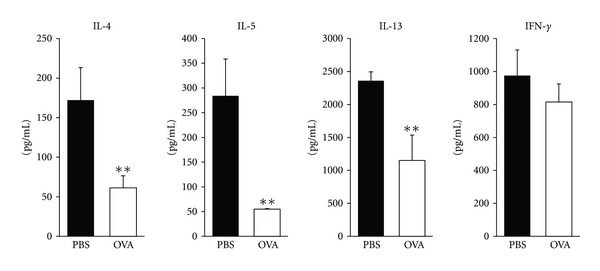
Th1/Th2 cytokine production from CD4^+^ T cells isolated from sublingually treated mice with allergic rhinitis. Culture supernatants of CD4^+^ T cells isolated from spleen of mice with allergic rhinitis were assessed for Th1 and Th2 cytokine production levels by ELISA. Representative results from three independent experiments containing three to five mice in each group are shown. Significance was evaluated by an unpaired *t*-test. **P < 0.01.

Given the suppressed antigen-specific IgE production observed in the serum of mice treated with preventive SLIT to nasally administered antigen, we hypothesized that Th2 responses would also be suppressed in these mice. To test this hypothesis, we compared the Th1/Th2 cytokine synthesis profiles of control and sublingually treated mice after chronic exposure to nasal allergens. Thus, Th1 and Th2 cytokine production was measured *in vitro* by OVA stimulation of CD4^+^ T cells isolated from spleen in nasally challenged mice. The amount of Th2 cytokines such as IL-4, IL-5, and IL-13 in culture supernatant was significantly lower in mice sublingually treated with OVA than in control mice (Figure 2). It is suggested that Th2 cytokine production in spleen was suppressed after sublingual administration of OVA.

### 3.3. Sublingual Administration of OVA Led to Upregulation of Foxp3- and IL-10-Specific mRNAs in CLN

**Figure 3:**
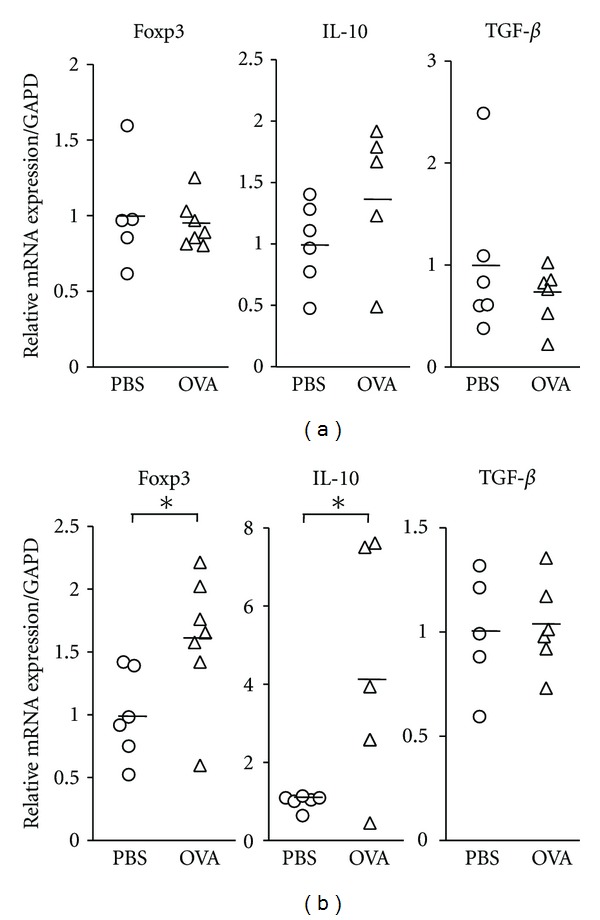
Messenger RNA expression of Treg-associated molecules in CD4^+^ T cells isolated from spleen and CLN of sublingually treated mice with allergic rhinitis. Messenger RNA expression of Foxp3, IL-10, and TGF-**β** in CD4^+^ T cells of spleen (a) and CLN (b) was determined by quantitative real-time PCR analysis. The expression of each molecule was normalized to the expression of GAPD. Each data was expressed as a ratio relative to mean expression level in PBS-treated control mice. Representative results from two independent experiments containing five to seven mice in each group are shown. Significance was evaluated by an unpaired *t-*test. *P < 0.05.

To further elucidate the immunopathological mechanisms underlying the suppressed Th2-mediated allergic responses observed in mice treated with preventive SLIT, we next set out to determine if the regulatory network formed by Tregs was altered by sublingual OVA treatment. CD4^+^ T cells isolated from the spleen and cervical lymph node and the regional LN of the upper airway were examined for the expression of Foxp3, a transcription factor of Tregs, and suppressive cytokines, IL-10, and TGF-**β**, by quantitative real-time PCR. Sublingual administration of OVA did not alter the mRNA expression pattern of Foxp3 and suppressive cytokines in the spleen (Figure 3(a)), but led to upregulation of Foxp3- and IL-10-specific mRNAs in CLN (Figure 3(b)).

### 3.4. Sublingual Administration of OVA Did Not Alter the Number of CD4^+^CD25^+^ Tregs, but Led to Upregulation of Foxp3- and IL-10-Specific mRNAs in Tregs of CLN

**Figure 4:**
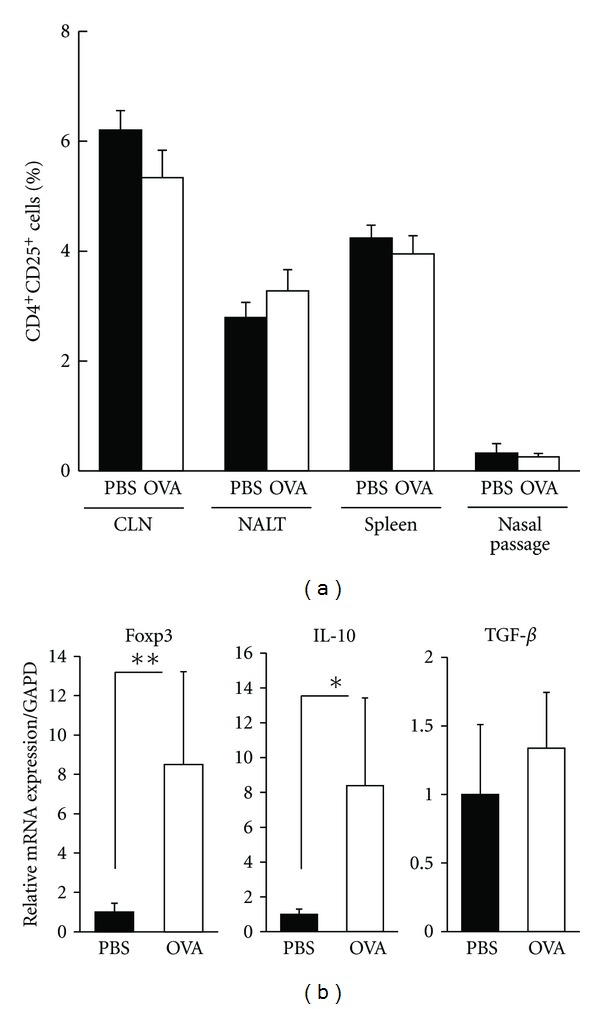
Flow cytometric analysis of Tregs and quantitative real-time PCR analysis of Treg-associated molecules in Tregs isolated from CLN of sublingually treated mice with allergic rhinitis. (a) The frequency of CD4^+^CD25^+^ Tregs in spleen, CLN, NALT, and nasal passage of sublingually treated mice with allergic rhinitis were determined and calculated using a flow cytometer. (b) Messenger RNA expression of Foxp3, IL-10, and TGF-**β** in CD4^+^CD25^+^ Tregs isolated from CLN of sublingually treated mice was determined by quantitative real-time PCR analysis. The expression of each molecule was normalized to the expression of GAPD. Each data was expressed as a ratio relative to mean expression level in PBS-treated control mice. Data are representative of two separate experiments. Significance was evaluated by an unpaired *t*-test. *P < 0.05 and **P < 0.01.

Thereafter, to determine the involvement of Tregs, we first examined the distribution and function of CD4^+^CD25^+^ Tregs, the representative population of immunosuppressive regulatory cells in the suppression of Th2-mediated allergic responses in sublingually treated mice with allergic rhinitis. There was no statistical difference in the total number of T cells (data not shown) or the frequency of CD4^+^CD25^+^ Tregs in the secondary lymphoid organs and nasal tissue between PBS- and OVA-treated mice (Figure 4(a)). Then, we performed quantitative real-time PCR analysis of Treg-associated molecules in Tregs isolated from CLN of mice treated with preventive SLIT in allergic rhinitis. Sublingual administration of OVA led to up-regulation of Foxp3- and IL-10-specific mRNAs in CD4^+^CD25^+^ Tregs in CLN (Figure 4(b)). These results indicate that sublingual administration of antigen may enhance suppressive function of CD4^+^CD25^+^ Tregs in CLN, even though it has no influence on the number of Tregs in such lymphoid tissue.

### 3.5. CD4^+^CD25^+^ Tregs in CLN of Sublingually Treated Mice with Allergic Rhinitis Strongly Suppressed Th2-Cytokine Production by Effector T Cells

**Figure 5:**
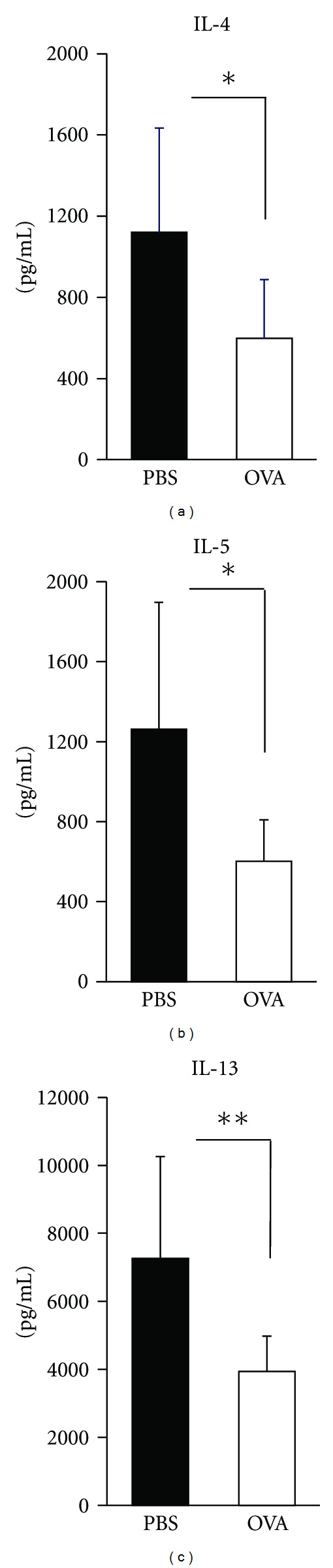
The impact of CD4^+^CD25^+^ Tregs in CLN of mice sublingually treated with antigen on Th2-cytokine production by effector T cells. CD4^+^CD25^−^ effector T cells isolated from CLN of mice which developed allergic rhinitis without having sublingual treatment were incubated with CD4^+^CD25^+^ cells isolated from CLN of mice sublingually treated with either PBS or OVA in the presence of feeder cells and plate-bound anti-CD3*ε* and soluble anti-CD28 antibodies for 96 hours. Th2-cytokine levels in culture supernatants were examined by cytokine ELISA. These data were obtained from two independent experiments containing three to five mice in each group. Significance was evaluated by an unpaired *t*-test. *P < 0.05 and **P < 0.01.

Because mRNA expression levels of Foxp3 and IL-10 in CD4^+^CD25^+^ Tregs in CLN of sublingually treated mice were significantly higher than in that of control mice, we next sought to determine whether the sublingual administration of antigen prophylactically would enhance the suppressive function of Tregs in CLN on Th2 cytokine production. When CD4^+^CD25^−^ effector T cells were incubated with CD4^+^CD25^+^ Tregs obtained from CLN of mice sublingually treated with either PBS or OVA, the production levels of IL-4, IL-5, and IL-13 in culture supernatant of effector T cells with CD4^+^CD25^+^ Tregs obtained from OVA-treated mice were significantly lower than those of effector cells with CD4^+^CD25^+^ Tregs of control mice (Figure 5). These data demonstrate that CD4^+^CD25^+^ Tregs in CLN are key players in the suppression of Th2 responses in sublingually treated mice with allergic rhinitis.

### 3.6. Sublingual Administration of Plasmids Encoding CCL19 DNA and CCL21-Ser DNA Together with OVA Suppressed Allergic Responses

**Figure 6:**
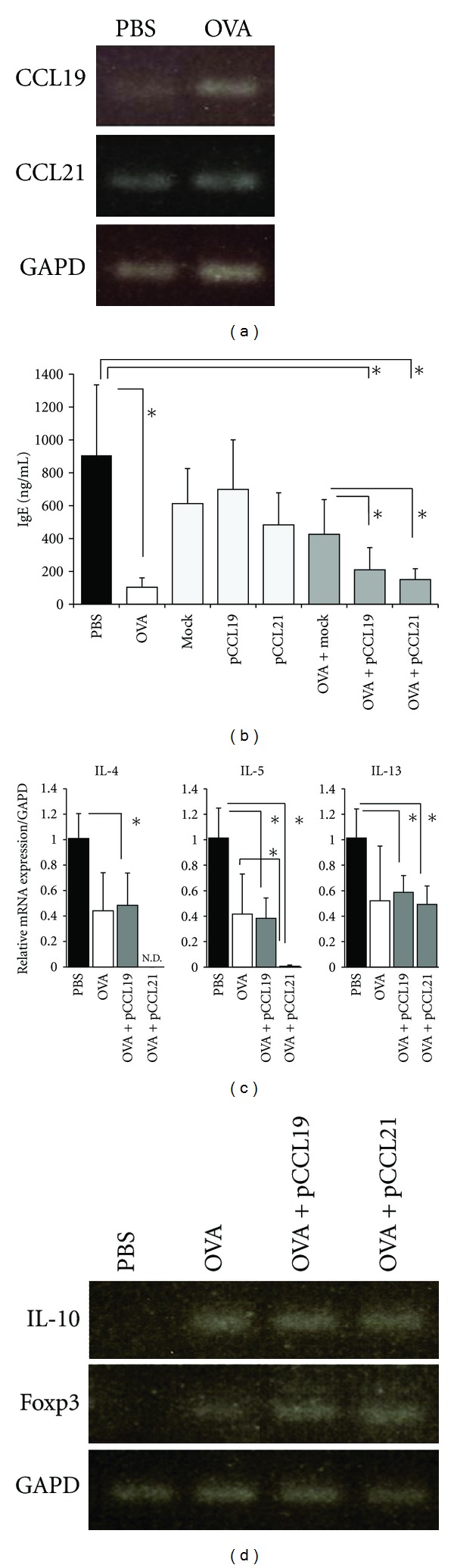
Chemokine expression in CLN of sublingually treated mice and the effect of sublingual administration of pCCL19/pCCL21 with antigen on Th2-mediated allergic responses. (a) Semiquantitative RT-PCR was performed to assess mRNA expression pattern of chemokines, CCL19 and CCL21 in whole cells isolated from CLN of mice sublingually treated with either PBS or OVA. (b) Mice were sublingually administered with either PBS, OVA alone, 100 *μ*g of mock DNA with or without OVA, 100 *μ*g of pCCL19 with or without OVA, and 100 *μ*g of pCCL21 with or without OVA for total three times before systemic sensitization and nasal challenge. OVA-specific IgE levels in serum were assayed by sandwich ELISA. (c) Messenger RNA expression of IL-4, IL-5, and IL-13 in CD4^+^ T cells isolated from spleen of mice sublingually treated with PBS, OVA, OVA plus pCCL19, and OVA plus pCCL21 was determined by quantitative real-time PCR analysis. The expression of each molecule was normalized to the expression of GAPD. Each data was expressed as a ratio relative to mean expression level in PBS-treated control mice. (d) Semiquantitative RT-PCR was performed to assess Foxp3- and IL-10-specific mRNA expression in CD4^+^ T cells isolated from CLN of sublingually treated mice. Data are representative of two separate experiments containing three to five mice in each group. Significance was evaluated by Kruskal-Wallis test (b, c). *P < 0.05. N.D. not detected.

Since the CCR7-CCL19/CCL21 pathway is crucial for the mobilization of immunocytes to sublingually administered antigen [11], we next sought to assess the involvement of these chemokines in preventive SLIT. To analyze the expression levels of mRNAs specific for CCL19 and CCL21-Ser in CLN, semiquantitative reverse transcription PCR was performed. Expression of the CCL19-specific gene was higher in the CLN of mice sublingually treated with OVA than in the CLN of PBS-treated control mice and the gene expression level of CCL21-Ser in CLN of OVA-treated mice was slightly higher than that of control mice (Figure 6(a)). Thereafter, we examined whether artificial addition of these lymphoid chemokines to mice using plasmid encoding CCL19 DNA (pCCL19) and CCL21-Ser DNA (pCCL21) would lead to the inhibition of allergic responses. OVA-specific IgE production in serum was suppressed by sublingual administration of OVA together with pCCL19 and pCCL21, respectively, although there was no significant difference in serum OVA-specific IgE levels between mice treated with OVA alone and mice treated with OVA plus these plasmids (Figure 6(b)). Indeed, OVA-specific IgE level was significantly lower in mice sublingually administered with OVA together with pCCL19 and pCCL21, respectively, than those administered with OVA together with mock DNA (Figure 6(b)). We next assessed mRNA expression of Th2 cytokine in the spleen of sublingually treated mice. The expression levels of mRNA specific for IL-4, IL-5, and IL-13 were lower in the spleens of mice sublingually administered with OVA plus pCCL19 and OVA plus pCCL21, respectively, than those of control mice (Figure 6(c)). Interestingly, IL-4- and IL-5-specific mRNA expression in mice treated with OVA plus pCCL21 were significantly lower than those of mice treated with OVA alone (Figure 6(c)). Thereafter, to determine the involvement of Tregs in the suppression of Th2-mediated allergic responses in mice treated with OVA plus pCCL19/pCCL21, we examined the number and frequency of CD4^+^CD25^+^ Tregs in spleen, CLN, NALT, and nasal mucosa. There was no statistical difference in the total number of T cells or the frequency of CD4^+^CD25^+^ Tregs in each tissue among four groups which consisted of mice treated with PBS, OVA, OVA plus pCCL19, and OVA plus pCCL21, respectively (data not shown). However, Foxp3- and IL-10-specific gene expression in CLN CD4^+^ T cells was detected in groups of mice treated with OVA alone, OVA plus pCCL19, and OVA plus pCCL21 at similar expression levels but not in PBS-treated group (Figure 6(d)).

### 3.7. Therapeutic Sublingual OVA Treatment after Induction of Allergic Rhinitis Suppressed Th2 Responses

**Figure 7:**
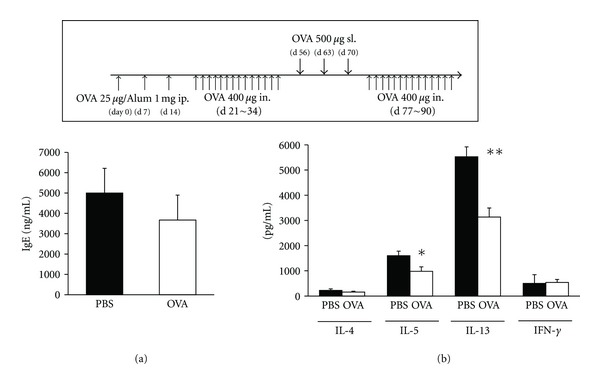
Antigen-specific serum IgE production and Th1/Th2 profile in spleen of mice which received therapeutic sublingual OVA treatment after induction of allergic rhinitis. Mice were sublingually administered with either PBS or OVA after intraperitoneal sensitization and nasal challenges with OVA. Thereafter, the mice received consecutive nasal challenges with OVA again and examined for their allergic responses. (a) OVA-specific IgE levels in serum were assayed by sandwich ELISA. (b) Culture supernatants of CD4^+^ T cells of spleen obtained from sublingually treated mice with allergic rhinitis were assessed for Th1 and Th2 cytokine production levels by ELISA. These data are representative of two independent experiments containing three to five mice in each group. Significance was evaluated by an unpaired *t*-test. *P < 0.05 and **P < 0.01.

In the clinical situation, sublingual treatment with allergen is normally performed after the patients were sensitized and exposed to nasal allergen and suffered from allergic rhinitis. Therefore, we also examined the efficacy of therapeutic sublingual OVA treatment in which mice were sublingually administered with OVA after they received systemic sensitization and local challenge of OVA and developed allergic rhinitis. There was no statistical difference in serum antigen-specific IgE levels between sublingually treated and control mice (Figure 7(a)), but production of IL-5 and IL-13 from splenic T cells was significantly suppressed in sublingually treated mice (Figure 7(b)). These results underline the efficacy of our experimental protocol as the murine model of SLIT in allergic rhinitis for the further examination.

## 4. Discussion

It has been proposed that SLIT is an effective therapy against allergic rhinitis in humans. Putative immunological mechanisms of SLIT are the induction of neutralizing antibodies with decrease in IgE/IgG4 ratio [17], immune deviation to Th1-dominant environment [18, 19], induction of antigen-specific CD4^+^CD25^+^Foxp3^+^ Tregs [7, 20], induction of Tregs producing IL-10 and/or TGF-**β** [19, 21, 22], a decrease in the recruitment of proinflammatory cells such as mast cells, basophils, and eosinophils in the airway mucosae [23], and the involvement of oral CD11b^+^CD11c^−^ macrophage-like cells in the differentiation of Tregs [24]; however, those are still under investigation. Besides, the amount of allergen needed in SLIT is 50 to 100 times more than that in SCIT [25], even though SLIT is considered to be safer than SCIT in terms of the appearance of anaphylaxis because of few proinflammatory cells (i.e., eosinophils and mast cells) in the submucosa of lingual tissue [26]. To address these issues, establishment of a murine model of SLIT and investigation of the mechanisms for the better targeting of the allergen to principal immune cells are necessary. We previously constructed an efficacious murine model of allergic rhinitis, in which systemically primed BALB/c mice showed nasal allergic symptoms such as sneezes and marked elevation of serum antigen-specific IgE level as well as Th2 cytokine production from CD4^+^ T cells in spleen and CLN after 14 consecutive nasal challenges with antigen [12]. In the present study, we could build up efficient murine models of preventive SLIT and therapeutic SLIT using 4 *μ*L of PBS containing OVA as a model antigen. Previous study demonstrated that FITC-labeled OVA remained exclusively located on the sublingual mucosa for 2 hrs and was undetectable in the esophagus and the intestine when the total volume of saline containing OVA sublingually applied was less than 10 *μ*L [13]. The decrease in antigen-specific IgE production and in Th2 responses could be observed in our experimental preventive model and these results agree with the previous findings reported by others [27]. In addition, a decrease in Th2 responses was observed even after therapeutic sublingual OVA treatment, though its mechanisms remain unknown. We have to acknowledge that there might be a different immunological mechanism involved in the suppression of allergen-specific responses between preventive SLIT and therapeutic SLIT in terms of tolerance induction by immune cells such as Tregs and DCs. Based on the above premise, we employed the preventive SLIT model for the purpose of shedding light on the target cells in the mucosal and systemic immune responses in the suppression of allergic responses.

Tregs play a central role in the regulation of autoimmune, infectious, and allergic diseases by cell-to-cell contact-dependent inhibition and by the secretion of anti-inflammatory cytokines such as IL-10 and TGF-*β* [28]. Th2 responses have been shown to be downregulated by naturally occurring CD4^+^CD25^+^ Tregs expressing forkhead/winged-helix family transcription factor P3 (Foxp3) and by inducible Tregs which secrete antigen-specific IL-10 [29, 30]. IL-10 and TGF-*β* decrease IgE production and inhibit the production of Th2 cytokines such as IL-4 and IL-5 [5]. In our present study, sublingual administration of OVA before systemic sensitization led to up-regulation of Foxp3- and IL-10-specific mRNAs, especially in CD4^+^CD25^+^ naturally occurring Tregs in CLN. Furthermore, Tregs in CLN of sublingually treated mice strongly suppressed Th2 cytokine production by effector T cells. Our data is supported by a recent finding in which not antigen-specific Tregs but naturally occurring antigen-nonspecific Foxp3^+^CD4^+^CD25^+^ T cells regulate allergic airway responses [31]. On the other hand, another recent study reported that IL-10-producing Tregs were induced after patients with pollinosis received SLIT [22]. From these findings confirmed by us and others, we can speculate that when exposed to an allergen, naturally occurring Tregs produce IL-10 or induce IL-10 production by other cells and trigger up-regulation of inducible antigen-specific Tregs, so that a more robust suppressive network is constructed. However, we need to further determine the Th2-suppression pathway by Tregs to assess whether IL-10 is directly involved or cell-to-cell contact-dependent inhibition is necessary.

In this study, OVA-specific IgG in serum did not increase even though IgE/IgG ratio decreased after mice received SLIT (data not shown). A decrease in the antigen-specific IgE/IgG4 ratio with an increase in IgG4 levels has been observed in a number of clinical SLIT studies with some exceptions in which no alterations in immune responses was detected during SLIT [25]. These controversial results might be caused by differences in the immunological nature of antigens as well as differences of IgG isotypes between rodents and human. Sublingual vaccination with inactivated or influenza virus to mice leads to effective protection against lethal virus infection through the induction of not only virus antigen-specific CD4^+^ and CD8^+^ T cells, but also antigen-specific serum IgG in a CD11c^+^ cell-dependent manner, despite no immune suppressive effects being induced through Tregs [32]. Moreover, while sublingual administration of OVA together with cholera toxin (CT) as mucosal adjuvant induces antigen-specific antibody responses as well as cytotoxic T cell responses [13], sublingual administration of OVA conjugated to the nontoxic B subunit protein of cholera toxin (CTB) induces antigen-specific Tregs and peripheral tolerance with increased TGF-*β* levels in serum [7]. Thus, it suggested that the mucosal immune system in the sublingual tissue discriminates the type or nature of antigen and adjuvant administered, leading to the modification of antigen presenting cells for the induction of whether tolerance or active antigen-specific immune response. 

Mucosal DCs play an important role in antigen detection as the front-line sentinels and in the recognition of the nature of antigen for the induction of appropriate immune responses. Chemokine network formed by CCR7 and its ligands CCL19/CCL21 are critically important for the migration of DCs in the skin and mucosal tissues such as gut into the draining lymph nodes for the induction of immune response [33]. Recent study showed that CCR7-CCL19/CCL21 signaling is responsible for the antigen uptake by DCs in the sublingual mucosa and the migration of those DCs into CLN [11]. In our present study, gene expression levels of CCL19 and CCL21 in CLN were higher in mice preventively treated with SLIT than in control mice (Figure 6(a)) and our data is in agreement with the previous findings. We formerly showed the regulatory role of CCL19 and CCL21 in allergic rhinitis [12]. In the murine allergic rhinitis model, the CCR7-CCL19/CCL21 signal is involved in the migration of CD4^+^CD25^+^ Tregs into CLN and nasal administration of plasmids encoding CCL19/CCL21 DNA leads to an increase of Tregs with an alteration of the DC profile in CLN, a decrease of serum antigen-specific IgE, and the suppression of allergic nasal symptoms. In this study, OVA-specific IgE production in serum was suppressed by sublingual administration of OVA together with pCCL19 and pCCL21, respectively, but not by sublingual administration of OVA together with the mock DNA (Figure 6(b)) in preventive SLIT. There was no statistical difference in OVA-specific IgE production between control mice treated with PBS and mice treated with OVA together with mock DNA. These data lead us to speculate that the empty vector alone might have adjuvanticity to enhance antigen-specific IgE production. The mRNA expressions of IL-4 and IL-5 were suppressed in the spleen of mice sublingually treated with pCCL21 together with allergen (Figure 6(c)). We still have not elucidated the mechanisms of limited suppression of the mRNA expressions of IL-4 and IL-5 and little decline in serum antigen-specific IgE level in mice treated with SLIT. One can speculate that Th2 cytokines and chemical mediators produced/degranulated by other immune cells, for instance, mast cells, basophils, and DCs may be involved in the phenomenon but further investigation will be needed. Taken together, these data indicate the possibility that the CCR7-CCL19/CCL21 pathway is involved in the suppression of allergic immune responses by regulating the migration of mucosal DCs and Tregs into CLN in SLIT; however, those mechanisms remain to be further elucidated. Moreover, the allergic responses may be suppressed even in the lower safety doze of antigen when sublingually administered together with pCCL19/pCCL21. Other recent studies demonstrated that CCR7 ligands have roles not only in the chemotaxis of the immune cells but also in the regulation of T cell responses by inducing cytokine production from DCs. CCL19 and CCL21 stimulate DCs to produce IL-23 [34], which enhances immunosuppressive function of Tregs [35]. 

In summary, the present study suggests the involvement of IL-10-expressing CD4^+^CD25^+^Foxp3^+^ Tregs of the draining LN in the suppression of Th2-mediated allergic responses in mice receiving SLIT. Further, suppression of allergic responses in the plasmid treatment enables us to suppose the presence of CCL19/CCL21-mediated regulatory mechanisms in the sublingual mucosa and provides the possibility of CCL19/CCL21 to be an adjuvant for a novel and more safe strategy of SLIT against allergic rhinitis.

## Abbreviations

SLIT: Sublingual immunotherapy
Tregs: Regulatory Tcells
Foxp3: Forkhead/winged-helix family transcription factor P3
CLN: Cervical lymph nodes
SCIT: Subcutaneous immunotherapy
NALT: Nasopharynx-associated lymphoid tissue
DCs: Dendritic cells
NP: Nasal passage
pCCL19: Plasmid encoding CCL19 DNA
pCCL21: Plasmid encoding CCL21-Ser DNA.
